# 3D-Printed Tablets of Nifurtimox: In Vitro and In Vivo Anti-*Trypanosoma cruzi* Studies

**DOI:** 10.3390/pharmaceutics17010080

**Published:** 2025-01-09

**Authors:** Giselle R. Bedogni, Ana Luiza Lima, Idejan P. Gross, Tatiana Prata Menezes, Andre Talvani, Marcilio Cunha-Filho, Claudio J. Salomon

**Affiliations:** 1 Institute of Chemistry Rosario, National Council for Scientific and Technical Research (IQUIR-CONICET), Rosario 2000, Argentina; gisellebedogni@gmail.com; 2 Laboratory of Food, Drug, and Cosmetics (LTMAC), School of Health Sciences, University of Brasilia, Brasília 70910-900, Brazil; anaaluiza.ln@gmail.com (A.L.L.); gross.idejan@gmail.com (I.P.G.); 3 Laboratory of Immunobiology of Inflammation, Biological Science Department/ICEB, Federal University of Ouro Preto (UFOP), Ouro Preto 35400-000, Brazil; tatiana.menezes@ufop.edu.br (T.P.M.); talvani@ufop.edu.br (A.T.); 4 Faculty of Biochemical and Pharmaceutical Sciences, National University of Rosario (UNR), Rosario 2000, Argentina

**Keywords:** nifurtimox, film casting, hot melt extrusion, 3D printing, fused deposition modeling, anti-*T. cruzi* activity

## Abstract

**Background/Objectives**: Chagas disease is a neglected tropical disease caused by infection with the parasite *Trypanosoma cruzi*. Benznidazole and nifurtimox are the only approved drugs for treating this condition, but their low aqueous solubility may lead to erratic bioavailability. This work aimed for the first time to formulate tablets of nifurtimox by hot melt extrusion coupled with 3D printing as a strategy to increase drug dissolution and the production of tablets with dosage on demand. **Methods**: Different pharmaceutical-grade polymers were evaluated through film casting, and those with promising nifurtimox amorphization capacity were further used to prepare filaments by hot melt extrusion. The printability of the obtained filaments was tested, and the polyvinyl alcohol filament was further used for printing tablets containing 120 and 60 mg of nifurtimox. **Results**: Three-dimensional tablets showed a remarkable improvement in the drug dissolution rate compared to commercial tablets and a dissolution efficiency 2.8 times higher. In vivo studies were carried out on Swiss mice. Parasitemia curves of nifurtimox printed tablets were significantly superior to the pure drug. Moreover, NFX 3D tablets provided a similar *Trypanosoma cruzi* reduction in plasmatic concentration to benznidazole, the gold-standard drug for acute-phase treatment of the Chagas disease. **Conclusions**: The findings of this work showed that hot melt extrusion coupled with 3D printing is a promising alternative for increasing nifurtimox biopharmaceutical properties and an attractive approach for personalized medicine.

## 1. Introduction

Nifurtimox (NFX) is a nitrofuran antiprotozoal drug prescribed to treat Chagas disease, which is transmitted by the vector *Trypanosoma cruzi* and affects both animals and humans. According to the Biopharmaceutical Classification System, NFX belongs to class II, exhibiting a low aqueous solubility while presenting satisfactory membrane permeability [1]. Controversially, this drug was also labeled as a class IV drug, indicating that limited permeability could be another concern [2]. This drug’s commercially available dosage form is an immediate-release tablet (Lampit^®^ 120 mg), which presents limited absorption and erratic bioavailability. Clinical use indicates that this dosage should be adjusted, particularly for pediatric patients who require 30 to 60 mg doses, as well as even smaller doses [1,2,3]. Consequently, novel delivery systems are urgently needed to improve their biopharmaceutical properties, providing a suitable treatment for Chagas disease.

Over the last few decades, hot melt extrusion (HME) has emerged as a promising technology in the pharmaceutical field. It involves the mixture of a drug and a polymer at a molecular level, affecting the crystalline state of the drug [4]. This increase in the amorphous phase of drugs and a reduction in particle size have helped improve the aqueous solubility of hydrophobic drugs [4,5,6].

The extrudate filaments obtained by HME are highly versatile and have multiple pharmaceutical applications, such as the preparation of an amorphous solid dispersion for solubility enhancement as granules or pellets, microencapsulation, taste masking, and the preparation of films, implants, semisolid formulations, capsules, and tablets, among others [6,7,8,9]. Recently, the use of HME filaments to feed three-dimensional (3D) printers has been explored, allowing us to obtain tablets with diverse geometries and personalized doses on demand [10,11,12]. In particular, the flexibility to produce customized doses of medications using this approach can be promising for Chagas disease, where patients exhibit a wide range with regard to age and weight, and part of the population may be undernourished [13].

Among the dozens of 3D-printing technologies currently available, the extrusion-based technique, particularly fused deposition modeling (FDM), is one of the most promising and most studied in the pharmaceutical field [14,15,16,17]. Briefly, FDM is based on the deposition of melted filament, where different layers bond with each other as they solidify after cooling, producing the desired dosage form. The small-scale production of personalized medicines can be achieved using industrially produced HME filaments in FDM 3D printers, enabling dose optimization for each patient during follow-up medical appointments. Moreover, FDM 3D printing makes it possible to safely combine various drugs and drug delivery in the same dosage form. Such an approach has been successfully applied with fluconazole, budesonide, hydrochlorothiazide, prednisolone, and acetaminophen, obtaining a wide range of final dosage forms, from controlled release to fast-release drug delivery systems [18,19,20].

During the HME process and the subsequent FDM 3D printing, the drug is exposed to high temperatures. Therefore, drug candidates must demonstrate thermal stability to ensure that no degradation will occur during the processing stages [21,22]. Recent studies have demonstrated that NFX is an excellent candidate for HME/FDM processes, since it is highly prone to remaining amorphous after melting and cooling without thermal degradation [7].

Thus, this work aimed to obtain, for the first time, NFX tablets by FDM 3D printing as an alternative to commercial tablets, allowing for personalized drug dosages and improving their bioavailability. For this purpose, several pharmaceutical polymers used in HME were tested by the film-casting method. Then, the thermal compatibility of NFX with the selected polymers was evaluated. Subsequently, NFX filaments were produced with compatible materials by HME, and the printable filaments were used to produce tablets using FDM 3D printing in two different dosages, 60 and 120 mg. The HME filaments and 3D tablets had their physicochemical properties evaluated, including their thermal and dissolution profiles. Finally, the Anti-*T. cruzi* activity of these innovative tablets was tested by an in vivo rodent model.

## 2. Materials and Methods

### 2.1. Materials

NFX (purity 99.9%, lot 160209), benznidazole (N-benzyl-2-nitro-1-imidazolacetamide), and Lampit^®^ (120 mg, lot SVF1381, Bayer S.A., Leverkusen, Germany) were kindly donated by Gador S.A. (Buenos Aires, Argentina) and Secretaria de Salud de la Nación (Buenos Aires, Argentina), respectively. Polyvinyl caprolactam–polyvinyl acetate–polyethylene glycol graft copolymer (Soluplus^®^, BASF, Ludwigshafen, Germany, Lot 84414368EO), polyvinyl alcohol (PVA, Parteck^®^ MXP, Merck, Darmstadt, Germany, Lot: F215B464), 60:40 linear random copolymer of N-vinyl-2-pyrrolidone and vinyl acetate (PVP/VA, Plasdone^®^ S-630, Ashland, Mumbai, Maharashtra, India, Lot 0002177615), hydroxypropyl methylcellulose (HPMC, Affinisol^®^, Colorcon, Harleysville, PA, USA, Lot 1099015561), hydroxypropyl cellulose (HPC, Klucel^®^ ELF, Ashland, Lot 40915), copolymers of ethyl acrylate, and methyl methacrylate with a low content of methacrylic acid ester with quaternary ammonium groups (Eudragit^®^ RLPO, Evonik, Essen, Germany, Lot 6170936626 and Eudragit^®^ RSPO, Evonik, Lot G0310398154) were donated. Triethyl citrate was obtained from Sigma-Aldrich (TEC, St. Louis, MO, USA). All reagents used were analytical grade.

### 2.2. Film Casting

NFX miscibility with different pharmaceutical grade polymers commonly used in extrusion processes and available in the laboratory was evaluated following the method described by Parikh et al., with modifications [23]. Briefly, NFX and polymer stock solutions were prepared with an equal final concentration. NFX was dissolved in acetone (30 mg mL^−1^ or 15 mg mL^−1^), and polymers (Soluplus^®^, PVP/VA, HPMC, HPC, Eudragit^®^ RLPO, Eudragit^®^ RSPO) were dissolved in a 90 % *v*/*v* acetone solution in water to facilitate polymer dissolution, reaching a final concentration of 30 mg mL^−1^. Due to solubility limitations, PVA was prepared in a 60% *v*/*v* acetone solution in water, with a final concentration of 15 mg mL^−1^. Specific volumes of each stock solution were mixed and placed in aluminum pans, reaching a final volume of 20 µL, to obtain the following NFX–polymer mass ratios: 20:80, 40:60, 50:50, 60:40, and 80:20. All samples were prepared and analyzed in duplicate. As a control, 20 µL of NFX dissolved in acetone was placed in an aluminum pan. Samples were kept at room temperature until they dried completely (around 24 h).

Differential scanning calorimetry (DSC) was performed in the range of 25 to 250 °C, with a heating rate of 5 °C min^−1^ under a N_2_ atmosphere (50 mL min^−1^) in a DSC-60 (Shimadzu, Kyoto, Japan). An empty pan sealed with a cover was used as a reference sample. The relative degree of crystallinity was calculated following Equation (1), where Δ_m_H was the melting enthalpy change from each sample corrected by NFX content and Δ_m_H_NFX_ was the melting enthalpy change in pure NFX (considered 100% crystalline).

(1) $$\mathrm{Degree}\;\mathrm{of}\;\mathrm{crystallinity}\;\left(\%\right)=\frac{\Delta_{m}H}{\Delta_{m}H_{NFX}}\times 100\;$$

### 2.3. Thermal Compatibility

Binary equimass physical mixtures of NFX and selected polymers were prepared (RSPO-pm, RLPO-pm, Soluplus-pm, PVP/VA-pm, and PVA-pm) and analyzed by thermogravimetric analysis (TGA) to obtain information about the stability of the samples from their decomposition profile. Samples were heated from 30 to 500 °C in platinum pans under a N_2_ atmosphere, with a heating rate of 10 °C min^−1^ (DTG-60H, Shimadzu, Kyoto, Japan).

### 2.4. Preparation of NFX Filaments by Hot Melt Extrusion

Physical mixtures of NFX with the selected polymers and plasticizer were prepared as described in Table 1. Mixtures were subjected to extrusion in a co-rotating conical twin-screw extruder (HAAKE MiniCTW, ThermoScientific, Waltham, MA, USA) operated in open mode at 50 rpm, and temperatures were chosen based on the polymer. The extruder was operated with a torque of 0.39 Nm, an extrusion speed of 1.74 g min^−1^, and a residence time of approximately 12 min. Filaments were ground and further sieved for characterization purposes to obtain a particle size range between 150 and 250 μm.

### 2.5. D Printing of NFX Dosage Forms

PVA-fil was produced by coupling the extruder outlet to a filament puller to produce filaments with a regular diameter of approximately 1.75 mm. These printable filaments were used to fabricate tablets with 120 and 60 mg of NFX using an FDM 3D printer (Voolt 3D model Gi3; São Paulo, Brazil). The templates were designed with Tinkercad^®^ (Autodesk^®^ Inc., San Rafael, CA, USA). The chosen geometry was cylindrical, with X = Y = 16.5 mm and Z = 6.5 mm for 120 mg tablets (PVA-3DP 120) and X = Y = 13 mm and Z = 5 mm for 60 mg tablets (PVA-3DP 60). All samples were printed with a needle with a diameter of 0.4 mm, using a 40 mm s^−1^ printing speed and 60% infill percentage. The layer thickness was set at 0.2 mm, and the tablets were printed using 33 layers. The printing temperature was 190 °C, and the build plate was 70 °C.

### 2.6. Tablets Characterization

Printed tablets were weighted using an analytic balance (AUW220D, Shimadzu, Kyoto, Japan), and their dimensions were measured using an electronic caliper. The results were expressed as the mean of 10 units. Drug content was determined by high-performance liquid chromatography (HPLC). Calibration curves were prepared in triplicate in the 2.5–20 μg mL^−1^ range for NFX (r^2^ = 0.999). Samples were analyzed by a Shimadzu chromatograph (Kyoto, Japan), model LC 20-AD, equipped with a DAD detector (SPD-20A), pump (LC-20D), degasser (DGU-20A3), automatic injector (model 9SIL-20AD), and oven (model CTO-20AS). The stationary phase was a C18 reversed-phase column (Shimadzu, 150 × 4.5 mm, 5 µm). The mobile phases consisted of 60% water and 40% acetonitrile using a gradient flow of 0.6 mL min^−1^ from 0.00 to 3.50 min and 0.5 mL min^−1^ from 3.51 to 12.00 min. The wavelength used for detection was 402 nm. The analysis of raw data and peak integrations were performed using the software LabSolutions v5 (Shimadzu, Kyoto, Japan).

### 2.7. In Vitro Dissolution Assay

A dissolution assay was performed in HCl 0.1 N to emulate the oral administration, with a bath temperature of 37 °C and a stirring speed of 75 rpm (299-8TS Ethik Technology, Sao Paulo, Brazil). At predetermined intervals, an aliquot of the dissolution medium (3 mL) was withdrawn and replaced by fresh medium pre-heated at 37 °C. Aliquots were adequately diluted, and their absorbances were measured at 396 nm (UV-1800, Shimadzu, Kyoto, Japan). NFX powder as supplied or the commercial tablet Lampit^®^ 120 were used as control. Assays were carried out in triplicate, and the results were expressed as mean  ±  standard deviation. Dissolution efficiency (DE) was calculated using Equation (2), where y is the percentage of dissolved product, and DE is the area under the dissolution curve between t1 and t2 expressed as a percentage of the curve at maximum dissolution y100, over the same period.

(2) $$DE=\frac{\int_{1}^{2}ydt}{y_{100}\times \left(t_{2}-t_{1}\right)}\times 100\;$$

### 2.8. In Vivo Anti-T. cruzi Activity Assay

#### 2.8.1. Animals and Parasites

Swiss female mice, aged 6–8 weeks, were obtained from the Animal Science Center at Universidade Federal de Ouro Preto. For the parasite, the Y strain of *Trypanosoma cruzi*, classified as *T. cruzi* II, was used in this study, with the strains obtained from successive passages in Swiss mice. The procedures adopted are under the ethical principles of animal experimentation preestablished by the National Council for Control of Animal Experimentation (CONCEA). This research was previously approved by the Ethics Committee on Animal Research of UFOP-CEUA (protocol n° CEUA n^o^.2151070922).

#### 2.8.2. Experimental Designs and Infection Procedure

Mice were randomly divided into eight groups of 6 individuals, resulting in 48 animals. Each animal was inoculated via intraperitoneal injection with 10^3^ blood trypomastigote forms of *T cruzi.* (Y strain). The 8 groups were divided according to the treatment: (i) infected, untreated mice; (ii) infected mice treated with benznidazole at a dose of 100 mg kg^−1^; (iii) infected mice treated with NFX at a dose of 25 mg kg^−1^; (iv) infected mice treated with NFX at a dose of 50 mg kg^−1^; (*v*) infected mice treated with NFX at a dose of 100 mg kg^−1^; (vi) infected mice treated with an NFX 3D-printed tablet at a dose of 25 mg kg^−1^; (vii) infected mice treated with an NFX 3D-printed tablet at a dose of 50 mg kg^−1^; (viii) infected mice treated with an NFX 3D-printed tablet at a dose of 100 mg kg^−1^. Treatment began 72 h post infection and continued for 27 consecutive days. Samples were administered orally (via gavage) in a volume of 0.1 mL, diluted in water suspended in 0.05% *w*/*v* methyl cellulose in appropriate concentrations for each dose. Parasitemia was monitored daily through an optical microscopy analysis of 5 μL blood samples obtained from tail bleeding, according to Brener’s method [24]. The area under the curve (AUC) was calculated to assess statistical differences. Additionally, daily animal behavioral observations were also performed.

## 3. Results and Discussion

### 3.1. Film Casting

Film casting is a popular approach to evaluate the capacity of different polymers to form a homogeneous phase with a drug after the complete evaporation of a common solvent for both components [25]. The interactions between the drug and polymer can strongly affect the crystallization behavior of the drug. Such interactions often inhibit solid-state crystallization by physically preventing the drug molecules from organizing into a crystal lattice [26]. In this context, such an effect on the crystalline fraction of the drug can be investigated based on the enthalpy change associated with the drug melting event [27,28]. Accordingly, polymers with different chemical structures and properties have been evaluated, including polymers with high Tg which can retard the recrystallization process from the amorphous state, increasing the stability [27].

Almost all polymers promoted a slight shift in the NFX melting peak of around 1.0 °C to lower temperatures, indicating low interaction. Except for Soluplus^®^, which had a 2.5 °C shift at the 50:50 and 60:40 ratios, and Eudragit^®^ RSPO, with a 5.0 °C shift at 50:50 ratio.

**Figure 1:**
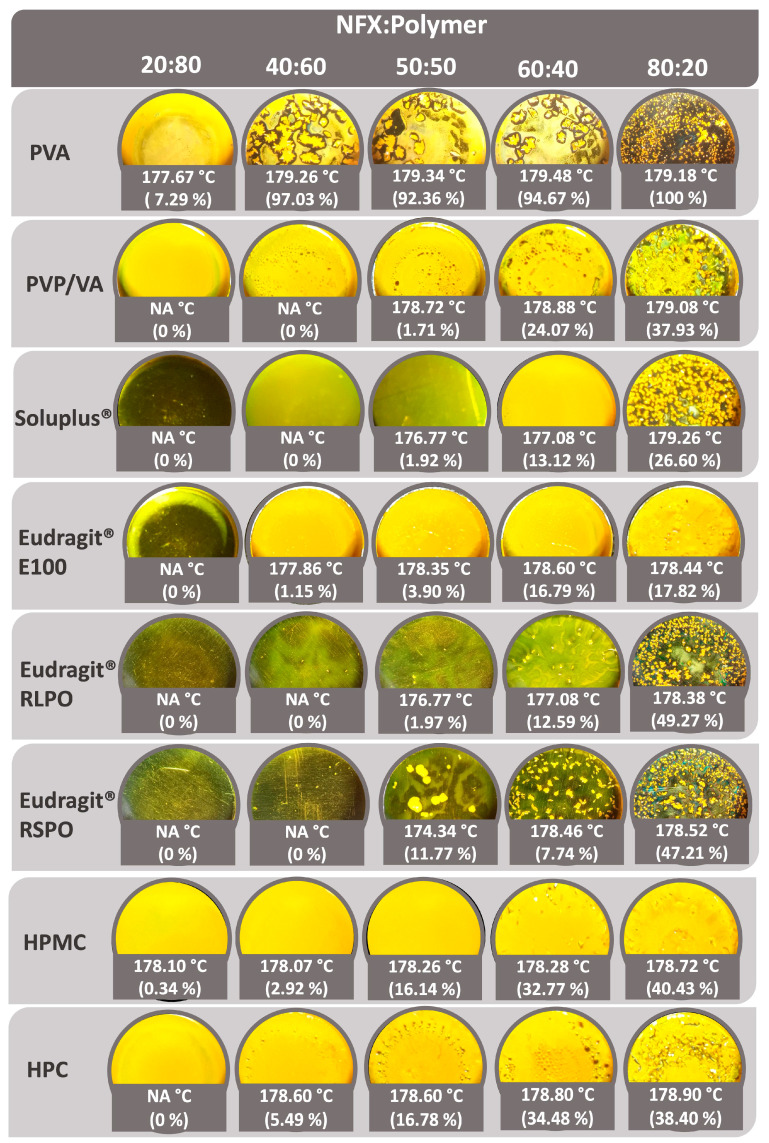
The film casting of pharmaceutical-grade polymers. Melting temperature peak and relative degree of crystallinity of NFX, expressed in °C and as a percentage, respectively, are shown. Images at 2× magnification. NA: not available.

In Figure 1, differences in the miscibility of NFX can be observed for different ratios of polymer used. For cellulose derivatives (HPMC and HPC), a homogenous dispersion of NFX was achieved with higher amounts of polymer. However, in samples with drug ratios of 50:50 or higher, a noticeable crystallization of the drug occurred, reaching around 40% of crystallinity in the 80:20 ratio.

Different acrylate polymers were evaluated, considering the Eudragit^®^ capacity to inhibit the recrystallization of drugs in supersaturated solutions [29]. With Eudragit^®^ RSPO and Eudragit^®^ RLPO, translucid films were obtained at a 20:80 ratio, with a complete disappearance of the endothermic signal attributed to NFX melting, which could indicate that 100% of NFX is in amorphous state. As the NFX ratio increased, some aggregates started to be observed with increased crystallinity percentages, reaching 49.27% and 47.21% for Eudragit^®^ RLPO and Eudragit^®^ RSPO, respectively. This capacity of both Eudragit^®^ variants to reduce the drug crystallinity has already been described for other hydrophobic drugs [30,31].

With Soluplus^®^ at 20:80, a translucid film was obtained that indicates the complete solubilization of NFX in the polymeric matrix. As the NFX proportion increased, films tended to lose their translucent characteristic until the formation of different drug aggregates. After 60:40, heterogeneities were formed in the recovered solid after solvent evaporation, and at 80:20, multiple aggregations of NFX were observed. Soluplus^®^ is an amphiphilic polymer that is expected to exhibit great potential regarding the amorphization of different drugs by reducing their fluidity in the polymer matrix or by establishing multiple hydrogen bonds [32,33].

Similar results were obtained with PVP/VA, but heterogeneities were visible as a result of the 50:50 ratio. Carbonyl oxygen from the PVP/VA tends to form molecular interactions with drugs containing hydrogen-bond donors [34]. This is not the case for NFX, since its chemical structure presents several hydrogen-bond acceptor functional groups, even though other possible weaker dipolar and dispersive interactions could occur.

PVA formed an opaque film at 20:80, with a drug crystallinity of 7.29%. Loss of homogeneity, with different degrees of aggregation, was obtained as the NFX ratio increased, achieving the total recrystallization of NFX at an 80:20 ratio. NFX recrystallization could have been induced by its low aqueous solubility and the water proportion used for dissolving PVA. Despite the experimental difficulty, PVA hydroxyls and hydrogen-bond acceptor groups of NFX should potentially interact, especially in high polymer proportions, which could inhibit drug self-association [35].

The higher drug amorphization values and better miscibility were used as selection criteria. Hence, Eudragit^®^ RSPO and Eudragit^®^ RLPO, Soluplus^®^, and PVP/VA were chosen for further evaluation. Although NFX tends to recrystallize with PVA during film casting, as stated before, this could have been a consequence of the solvent mixture used to dissolve the polymer rather than the drug–polymer interaction potential. Therefore, considering its rapid aqueous dispersion and high-quality properties for 3D/FDM printing with drugs, PVA was also added in the next phase of the study [36].

### 3.2. Thermal Compatibility

**Figure 2:**
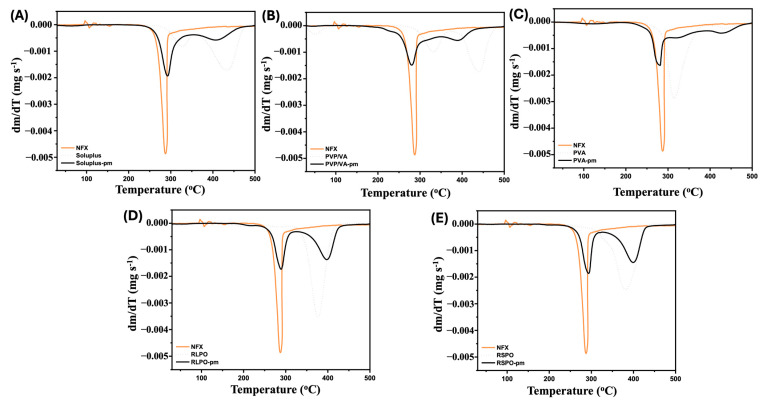
The thermogravimetric analysis of the drug and polymer as supplied, and their respective physical mixtures (pm) for thermal compatibility evaluation, namely (**A**) Soluplus-pm; (**B**) PVP/VA-pm; (**C**) PVA-pm; (**D**) RLPO-pm; (**E**) RSPO-pm.

NFX, as supplied, presented a mass loss pattern similar to other nitrofuran derivatives [37]. As seen in Figure 2, this drug exhibited a first intense decomposition step between 283.1 °C and 291.4 °C with a 48.37% mass loss. In addition, a gradual mass loss of 16.39% occurred after 292 °C, reaching a total mass loss of 64.76%. These results are in agreement with previously published results [7].

The TGA of Soluplus-pm showed a decomposition that occurs in two steps: the first at 290.8 °C, with a mass loss of 43.81%, and another one at 401.4 °C, with a mass loss of 31.05% (Figure 2A). Similar mass loss profiles were obtained by Kulkarni et al. and Al-Akayleh et al. with physical mixtures of morin hydrate and curcumin and Soluplus^®^ [38,39]. PVP/VA-pm, in turn, showed a maximum decomposition at 296.6 °C, losing 68.79% of its mass (Figure 2B). For PVA-pm, a total mass loss of 87.67% that occurred in two steps was observed (Figure 2C). The initial mass loss was 17.33% between 157.4 °C and 159.5 °C, followed by a more pronounced loss between 285.7 °C and 412.2 °C, relative to 70.34%.

Eudragit^®^ polymers have shown promising results for 3D-printing solid dosage forms due to their thermal stability [40]. As shown in Figure 2, both polymers presented a decomposition profile in three steps. RLPO-pm had a smooth mass loss at 208.5 °C (13.21%) and at 290.0 °C (24.97%) (Figure 2D). RSPO-pm showed a similar profile, with an initial mass loss of 15.32% at 253.9 °C, and 22.29% at 299.9 °C (Figure 2E).

The expected decomposition profiles appear preserved in the mixtures with all the polymers tested, especially the first degradation event, which corresponds to NFX and is more relevant for the sample stability (Figure 2). Moreover, the initial decomposition temperatures of each sample remained at the same level, showing that the drug–polymer mixtures are compatible. Finally, the decomposition ranges of the mixtures are above 200 °C, which makes them suitable for the HME process and FDM 3D printing [41,42].

### 3.3. Preparation of Filaments Loaded with NFX

Temperature is a key factor for HME, since it needs to be high enough to reach an appropriate viscosity that facilitates the mixture of the drug into the polymer matrix and its flow through the equipment, but not too high, as that could lead to the thermal degradation of the drug, or the cleavage of the polymer chain [43]. A plasticizer was added to the extrusion process to ensure its viability. The plasticizer selection for each polymer was based on the available literature and previous tests carried out in our laboratory [19,44,45].

**Figure 3:**
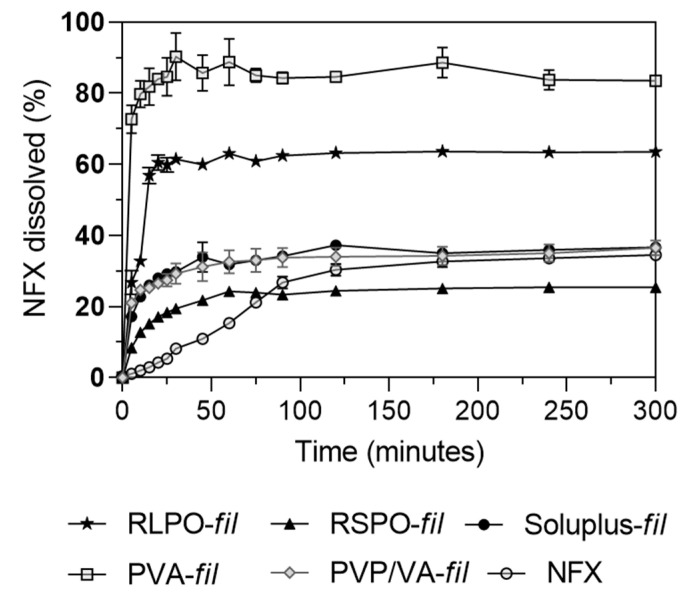
The in vitro dissolution profiles of filaments prepared by HME. Raw NFX was used as control. Data are represented as the mean of *n* = 3.

As seen in Figure 3, PVA-fil had a higher dissolution rate, dissolving more than 80% of NFX within the first 20 min. In the same period, 60% was dissolved from RLPO-fil, while 26%, 21%, and 17% from Soluplus-fil, PVP/VA-fil, and RSPO-fil, respectively. These results are quite impressive compared with the dissolution profile of NFX as supplied, in which only 4% was dissolved at 20 min, reaching a maximum dissolution of around 30% after 120 min.

Eudragit^®^ RSPO and RLPO are polymers commonly used for retarded release [46]. The difference between both is related to the higher permeability of Eudragit^®^ RLPO compared to Eudragit^®^ RSPO, which could allow RLPO-fil to swell faster than RSPO-fil, therefore facilitating the diffusion of NFX from the matrix [47]. The release mechanism in the PVA matrix also involves swelling-dependent diffusion, but PVA-fil showed a fast release profile [48]. In contrast, Soluplus-fil and PVP/VA-fil showed a slower release rate, which could be due to a slower process of matrix disintegration and formation of polymeric micelles [49].

From DSC data, Soluplus-fil had 14% NFX in a crystalline state, while PVA-fil and PVP/VA-fil had 21% and 23%, respectively. No endothermic event was observed for RSPO-fil and RSPO-fil, suggesting a total amorphization of NFX. These changes in crystallinity do not appear to be directly related to the different dissolution rates observed, which are more dependent on the polymer disintegration behavior [50]. Based on the outstanding dissolution performance of PVA-fil and its excellent printing characteristics, it was chosen to produce 3D-printed tablets.

### 3.4. NFX 3D Tablets

**Figure 4:**
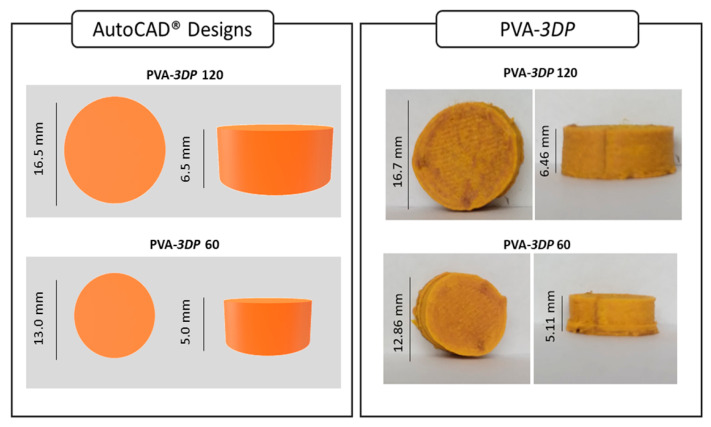
Design and microphotographs of obtained 3D-printed tablets.

Extrudate filaments for FDM 3D printing must be plastic enough to be feedable and avoid breaking during the feeding process, while not being so soft that they cannot be pushed through the equipment [51,52,53]. Accordingly, PVA-fil met the printing requirements, and cylindrical tablets were successfully printed (Figure 4). For 120 mg printed tablets (PVA-3DP 120), the measured dimensions were 16.70 mm (±0.43) × 6.46 mm (±0.07), and the average weight was 1.25 g (±0.03). The 60 mg printed tablets (PVA-3DP 60) had a diameter of 12.86 mm (±0.60) and 5.11 mm (±0.10) thickness, with an average weight of 0.64 g (±0.04).

HPLC analysis presented recovery percentages of NFX of 92.43% (±6.94) for the 60 mg tablets and 109.45% (±10.07) for the 120 mg tablets. No sign of degradation was observed, i.e., the NFX peak remained unaltered after HME and FDM treatment at the same retention time. These results confirmed the potential of 3D printing for precise dosage adjustment based on printer settings.

**Figure 5:**
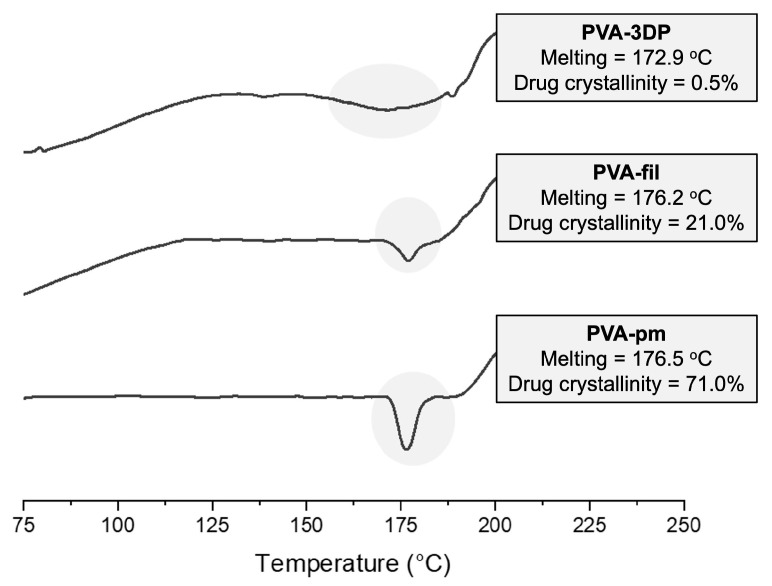
DSC curves of samples at different production stages, PVA-pm, PVA-fil, and PVA-3DP.

A thermal characterization of the tablets was performed, as shown in Figure 5. The drug crystallinity is reduced even in the physical mixture (PVA-pm = 71.02%), due to an interaction effect of the drug with the formulation during the heating of the DSC analysis [54]. The extrusion process, in turn, as previously mentioned, produced an extensive reduction in the drug crystallinity in PVA-fil (21.04%). The second heating of the sample after the 3D-printing process accentuated the degree of amorphization of NFX, which reached almost total sample amorphization. In this case, the higher degree of drug amorphization is possibly caused by additional heating to higher temperatures.

**Figure 6:**
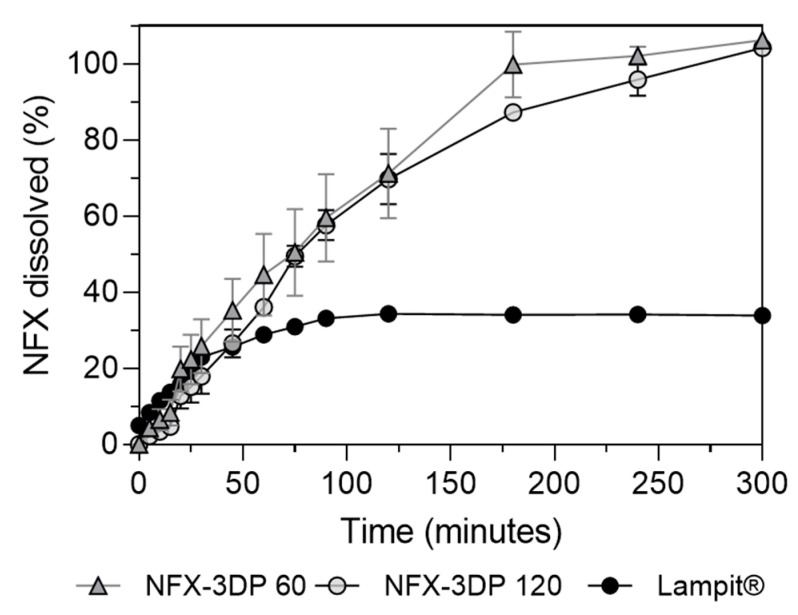
The dissolution profiles of printed tablets of NFX of 120 mg and 60 mg and the commercial tablet Lampit.

The differences observed in the dissolution profiles between PVA-fil from Figure 3 and NFX-3DP 60 and NFX-3DP 120, presented in Figure 6, can be attributed to the wettability and increased surface area presented by PVA-fil, since it was used as powder. A commercial NFX tablet (Lampit^®^ 120 mg) was used as the control. For the first 45 min, Lampit^®^ and PVA-3DP 120 had similar dissolution rates, while PVA-3DP 60 was slightly higher. Marked differences between printed and commercial tablets can be appreciated after 60 min, where Lampit^®^ reached a maximum of 34% while the 3D tablets dissolved nearly 100% of the drug after 300 min.

Both PVA-3DP 60 and PVA-3DP 120 3D tablets presented similar dissolution profiles, as observed by the dissolution efficiency (DE) analysis (Table 2), demonstrating that the printing process can adjust the dosage without altering its dissolution profile. The comparison at 30 min (DE_30_) of PVA-3DP 120 and Lampit^®^ indicates a similar value, which is also evident in Figure 6 where both profiles are overlapped. Higher differences are observed after 120 min, with a DE120 of 28% and 15% for PVA-3DP 120 and Lampit^®^, respectively, and 47% and 17% after 300 min (DE_300_).

Dissolution profiles were modeled with different mathematical models to determine the dissolution mechanism involved in the printed tablets (Table 3). The determination coefficient (R^2^) values suggested that the first-order model fit better for the evaluated tablets. This implicates a dissolution dependent on NFX concentration in polymeric films or porous matrixes, where dissolution rates decrease as the gradient concentrations decline in time [55]. Korsmeyer–Peppas also showed a good correlation for the PVA-based 3D-printed tablets, which indicates anomalous transport, where the mechanism of drug release is governed by diffusion and swelling due to the slowly rearranged polymeric chains.

### 3.5. In Vivo Antichagasic Activity Assay

**Figure 7:**
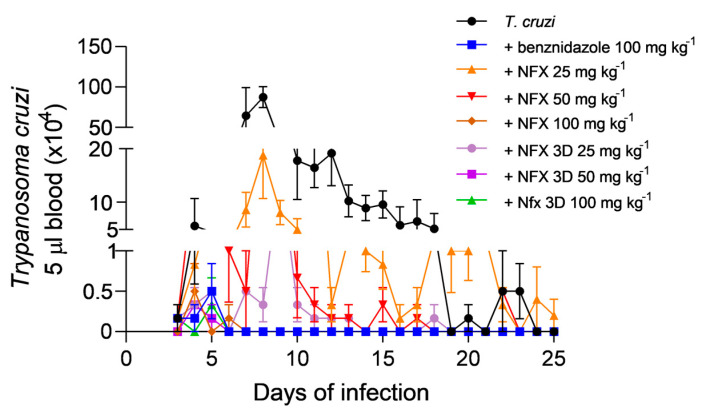
Parasitemia curves in mice infected by *T. cruzi* treated with benznidazole, NFX, or 3D-printed NFX tablets. The curve represents the number of circulating parasites in Swiss mice over a 25-day infection period. Each treatment group consisted of six animals.

Animals treated with the 3D tablet showed no signs of sociability disturbances, fur bristling, diarrhea, changes in food intake, or any other symptoms indicative of central nervous system toxicity associated with the 3D drug administration. The parasitemia curve demonstrates the efficacy of different treatment regimens of NFX in comparison to benznidazole against *T. cruzi* infection. The control group, which received no treatment, exhibited a high peak of parasites in the blood around day 7 post infection. In contrast, all treatment groups showed a significant reduction in parasitemia levels. The benznidazole-treated group (100 mg/kg) maintained parasitemia levels close to zero throughout the study period, demonstrating high efficacy, which is expected for this drug in a high-dose regime (Figure 7).

Among the NFX treatments, the pure drug formulations showed dose-dependent efficacy, with higher doses resulting in lower parasitemia levels. Furthermore, the NFX 3D tablets at doses of 25 mg/kg, 50 mg/kg, and 100 mg/kg demonstrated consistent and effective suppression of *T. cruzi*, comparable to the benznidazole-treated group (Figure 7). Notably, the NFX 3D tablets exhibited enhanced efficacy compared to the pure drug, even at equivalent doses. AUC analysis revealed a highly significant difference (*p* < 0.0001) between untreated infected animals and all treatment groups. When comparing the benznidazole (100 mg/kg) curve to other therapies, the NFX 25 mg/kg and NFX 50 mg/kg treatments showed statistically significant differences (*p* < 0.001). The processing of NFX by HME and 3D FDM decisively modified its physicochemical properties. Indeed, the conversion of this drug to the amorphous state and the high degree of interaction with the water-soluble polymer matrix promoted important improvements in its oral pharmacokinetics. This underscores the potential of such technology in enhancing therapeutic outcomes of existing drugs, offering an encouraging alternative for treating Chagas disease.

Indeed, Chagas disease is a neglected disease, and there is a lack of research on drug delivery involving NFX. Recently, an association between benznidazole and NFX in self-nano emulsified drug delivery systems impregnated on mesoporous silica particles was proposed, with a rapid dissolution rate and enhanced reduction in parasitemia [56]. The approach presented in our work is a more straightforward and lower-cost technological solution, which are crucial features in this case.

## 4. Conclusions

This work describes the successful application of HME coupled with FDM 3D printing to produce NFX tablets. Printed tablets presented a remarkable improvement in the NFX dissolution profile compared to commercial tablets. Such a result could be explained by the amorphization of NFX in the PVA matrix and mainly by the rapid dissolution profile of this polymeric matrix. Moreover, the 60 mg tablets showed a dosage accuracy and similar performance to the 120 mg tablets, validating 3D printing as an encouraging technology for personalizing antichagasic therapy. The study reinforces that the association of industrial production of filaments by HME to feed FDM 3D printers aimed at printing personalized recipes can solve the clinical needs of diversified dosages for treating Chagas disease.

Finally, the parasitemia curves in the in vivo studies showed that the NFX 3D tablets presented a superior antichagasic activity compared to the pure drug. Additionally, the NFX 3D tablets showed similar *T. cruzi* reduction in plasmatic concentration to benznidazole, the gold-standard drug for acute-phase treatment of the Chagas disease. It is crucial to monitor the thermal processing conditions carefully. Furthermore, the long-term stability of the formulation needs to be studied in further studies. Nevertheless, such a performance improvement had never been demonstrated before for NFX, which once again positions this molecule as a promising option for treating Chagas disease.

## Figures and Tables

**Table 1:** Description of physical mixtures prepared by HME.

	Soluplus-Fil	PVP/VA-Fil	RSPO-Fil	RLPO-Fil	PVA-Fil
Composition % (m/m)	50% Soluplus^®^ 40% NFX 10% TEC	60% Plasdone^®^ 40% NFX	70% Eudragit^®^ RSPO 20% NFX 10% TEC	70% Eudragit^®^ RLPO 20% NFX 10% TEC	80% Parteck^®^ MXP 10% NFX 10% TEC
HME parameters	110 °C 50 rpm	110 °C 50 rpm	120 °C 50 rpm	120 °C 50 rpm	150 °C 50 rpm

**Table 2:** Dissolution efficiency calculated for 3D-printed tablets and Lampit^®^.

DE	PVA-3DP 60	PVA-3DP 120	Lampit^®^ 120 mg
DE_30_	12.89	8.98	9.79
DE_60_	22.32	18.01	12.80
DE_120_	29.77	28.79	15.45
DE_240_	49.90	43.62	17.19
DE_300_	51.04	47.90	17.04

**Table 3:** Kinetic parameters from different mathematical models.

Model	Parameter	NFX-3DPT 60	NFX-3DPT 120	Lampit^®^ 120 mg
First order (with Fmax)	K_1_	0.005	0.006	0.026
	F_max_	187.67	136.48	33.32
	R^2^	0.9919	0.9800	0.9919
Higuchi	K_H_	6.823	6.218	2.51
	R^2^	0.9141	0.8890	0.7790
Korsmeyer–Peppas	K_HP_	3.46	2.329	5.44
	*n*	0.63	0.728	0.34
	R^2^	0.9609	0.9478	0.9152
Hixson–Crowell	K_HC_	0.003	0.003	0.000685
	R^2^	0.9274	0.9703	−0.1194

## Data Availability

The raw data supporting the conclusions of this article will be made available by the authors upon request.
